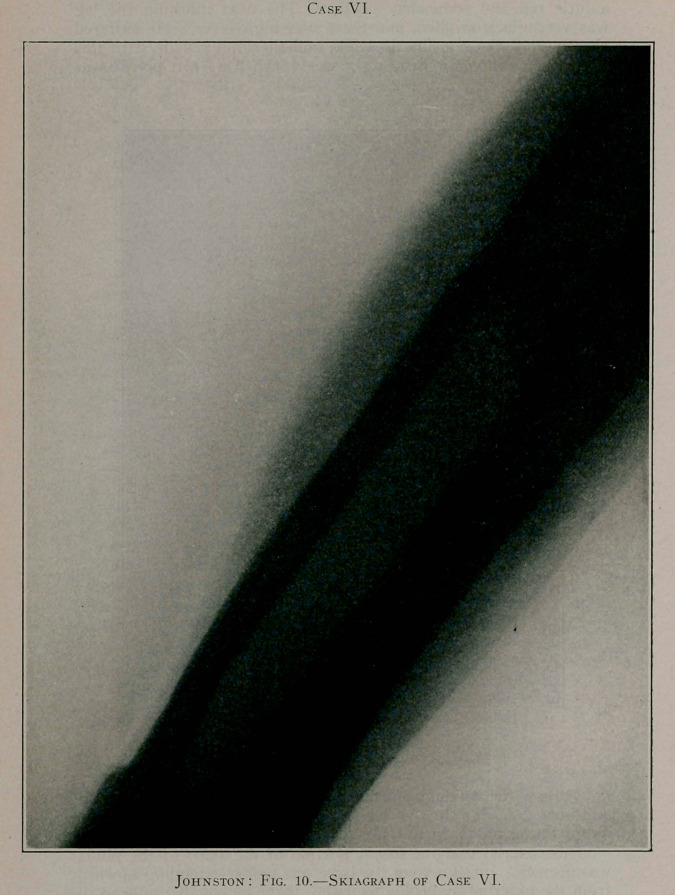# Complete Removal of the Shaft of the Tibia for Osteomyelitis, with Restoration of the Bone1Read at the annual meeting of the American Surgical Association at Saint Louis, Mo., June 16–18, 1904.

**Published:** 1905-01

**Authors:** George Ben Johnston

**Affiliations:** Richmond, Va., Chief of staff Memorial Hospital, Fellow of the American Surgical Association.


### BUFFALO MEDICAL JOURNAL.

VOL. XLIV.—LX. JANUARY, 1905. No. 6

### ORIGINAL COMMUNICATIONS.

### Complete Removal of the Shaft of the Tibia for Osteomyelitis, with Restoration of the Bone.[1]

BY GEORGE BEN JOHNSTON, M. D., Richmond, Va.,
Chief of staff Memorial Hospital, Fellow of the American Surgical Association.

THE following six cases of removal of the entire shaft of the tibia or fibula are presented because the results of operative treatment were so satisfactory, restoring otherwise hopelessly damaged limbs to usefulness.

Osteomyelitis is a disease which occasions much suffering, produces many deformities, and causes many deaths. It is therefore of sufficient importance to warrant a word of warning to physicians and ask on their part early recognition of it and prompt reference to a surgeon.

It is not meant to review the subject of osteomyelitis to this body of surgeons, but merely to present these cases and briefly make a few suggestions to such physicians as may see this report. Some forms of osteomyelitis are so obscure as to be difficult of recognition, and great damage is often done before a correct diagnosis is made and surgical treatment practised. The milder forms are mistaken for "growing pains," malarial fever, typhoid fever, and rheumatism; the more violent and active, for erysipelas.

When it is remembered how destructive this malady is and how dangerous to limb and life, the importance of early diagnosis and prompt reference to a surgeon are manifest.

The infection is not always of the same degree of virulence. The milder forms may pursue a more or less chronic course, but

1. Read at the annual meeting of the American Surgical Association at Saint Louis, Mo., June 16–18, 1904.

the acuter forms are so violent as often to cause prompt death or at least to destroy a bone, and this in a few hours.

The diagnosis is commonly not difficult. It generally attacks young, growing children, usually males. The bones oftenest involved are the exposed ones, notably the tibia. It is almost invariably traced to an injury, which is sometimes too trivial to be noticed at the time it is inflicted.

The constitutional manifestations vary all the way from malaise, general indisposition, and slight fever to a profound, overwhelming, and even fatal septicemia.

Where infection is slight the course of the trouble is mild and more or less obscure. Where the infection is virulent the symptoms are violent.

The age and sex of the patient, the history of an injury, the character of the pain, generally worse by night, a tender spot in the course of a bone, with redness and swelling, if superficial, suggest osteomyelitis.

Treatment must be prompt. Free drainage is imperative. If the disease involves only the superficial aspect of the bone, a free incision with proper disinfection and maintained drainage may be all that is required. Should the infection be in the medullary cavity or be otherwise deep seated, it must be found and proper drainage established. If the lesion has advanced to complete destruction of the bone, the treatment must be sufficiently radical to encompass the removal of all dead bone. This will occur in the more violent forms unless treatment is resorted to early enough to destroy the infection and thus prevent complete destruction. Where the major portion of a bone's shaft is destroyed, or is so involved as to require removal, this must be done. Where the entire shaft is removed, regeneration can only be of periosteal origin, and therefore the periosteum must be carefully preserved.

After free incision and removal of all diseased or dead bone, the wound should be most carefully antisepticised and maintained in this condition. Throughout, immobilisation is important and gives much comfort. For this purpose an ordinary fracture-box is the best appliance.

The dressings are to be as infrequent as is consistent with asepsis and always gently done. As new bone tissue begins to form, the parts may be shaped by the proper adjustment of adhesive strips and bandages, so that deformity may be lessened. When cicatrisation is complete, a light plaster-of-Paris cast is applied for the purpose of affording protection to the young and tender bone. The body weight should not be borne on the limb until the new bone has attained sufficient rigidity to support it safely. The general health will always require attention. Tonics, ample food, and fresh air will expedite recovery.

Where a disk of bone can be left covering the epiphyseal line, no deformity in the length of the bone will result. If the epiphyseal junction is destroyed, there will necessarily occur shortening. This may mean aggravated deformity, but no great impairment of limb function.

When one of a pair of companion bones is destroyed, the other invariably takes on compensatory hypertrophy.

Case I.—1888. T. R., a boy, aged 7 years, had an injury to the right tibia, falling against a curbstone. Seven days after a painful swelling appeared over the lower third of the left shin. The family treated him for rheumatism, and not until the symptoms became alarming was a physician called. The family physician treated him with poultices, quinine, and opium for three weeks. I saw him in October, 1888, when the disease had existed over six weeks. He was much run down in health and very septic. The swelling was immediately opened by a free incision. It was discovered that the tibia was dead and the periosteum detached from an inch above the junction of the lower end of the shaft with the epiphysis to a point two inches below the upper epiphyseal attachment. The dead portion of bone was removed with a thin disk of healthy bone attached. This was accomplished by stripping away the periosteum where it was attached in small islands to the diseased portion of the bone, and separating it from the healthy bone a short distance above and below the diseased portion, and then with a chisel dividing the shaft in healthy bone and lifting the shaft thus separated out of its bed. The periosteum was intact throughout. An antiseptic dressing was applied after disinfecting the enormous wound thus made, and the dressed leg was placed in an ordinary fracture-box.

Almost immediately the child's general condition improved, all pain subsiding and fever disappearing, and nutrition was resumed.

The wound granulated rapidly, and in the course of three weeks a needle could be made to indicate the presence of bone tissue in the granulations. Three months were required for the new bone to form and the wound to cicatrise completely. When this was accomplished the leg was encased in a plaster-of-Paris splint and the boy allowed to go on crutches. He was not permitted to use the limb for a year, at the end of which time he walked freely without limp or pain. The new tibia was somewhat ill-shapen, being flattened from before backward, but had sufficient strength to meet all requirements.

I was able to keep track of this little fellow for six years after the removal of the shaft of his tibia. During this time he seemed perfectly well and suffered no inconvenience from the loss of his bone, the function of the leg being perfect.

I regret I have no photographs of this case.

Case II.—1894. T. S., a boy, aged 8 years, while playing ball was injured on the right shin, the trouble being so trifling at the moment as to receive no attention either from the boy or

CASE II.

JOHNSTON: FIG. 1.—AFTER OPERATION.

his parents. A week later he began to complain of a deep-seated pain in the shin, aggravated at night; had high fever, loss of appetite, and rapid wasting of flesh. When these symptoms had progressed a few days I was consulted.

A diagnosis of osteomyelitis was made and surgical treatment applied. Exposure of the tibia displayed a deeply congested and thickened periosteum. The medullary cavity was trephined

Case II.

Johnston: Fig. 2.—Skiagraph.

and a considerable quantity of fetid pus evacuated. The symptoms were not promptly or materially ameliorated by this treatment. The disease rapidly spread, so that in ten days after the trephining of the bone a second operation was demanded and a longer incision made, extending two inches below the knee and well down to the ankle. It was found that about four-fifths of the shaft of the tibia were completely dead, the periosteum being separated almost entirely from this portion of the bone. The dead portion of the bone was removed, comprising about four-fifths of the shaft, the division being made well out into healthy bone. The after-treatment was the same as applied in the former case, including immobilisation of the leg in a fracture-box.

This youth made a rapid recovery, and the result in his case is absolutely perfect, a skiagraph showing practically no deformity in the tibia. He has grown almost to manhood, is leading an active life, and has positively no lameness, the two limbs being the same length and apparently of the same strength.

Case III.—R. M., a boy, aged 13 years, in December, 1893, received a blow on the left ankle too insignificant to attract attention. Three days after this injury a painful, red swelling appeared just above the joint on the tibial aspect of the leg. This at first was thought to be rheumatism. It extended up the leg so rapidly, and the redness and swelling increased so that it was later thought to be erysipelas. I saw him five days after the appearance of the swelling. His pain was excruciating. He was overwhelmed with sepsis, and his condition appeared most precarious. The diagnosis of acute infectious osteomyelitis was made and no time lost in opening the pus cavity. An incision was made the whole length of the tibia and an enormous amount of stinking pus poured out. Everywhere the periosteum was detached, and likewise the epiphyseal lines were separated, so that the entire shaft of the bone was found literally floating in a pus sac. The bone was lifted out without the application of a particle of force or the stroke of a knife to free a single attachment.

After proper preparation of the wound it was packed with iodoform gauze and outside dressings applied. The leg was put into an ordinary fracture-box and immobilised by packing about it snugly a quantity of wheat bran. For many days the issue was doubtful, but finally improvement began and progressed steadily. The leg was kept in a fracture-box until the wound had healed and new bone formed, after which time a light plaster-of-Paris dressing was applied and the boy allowed to go on crutches. At the end of eight months the new bone was deemed strong enough to support the weight of the body. On account of the destruction of the epiphyseal lines there was considerable deformity in this case, the tibia not growing in length. Nevertheless the limb is perfect in function, and when a thick-soled shoe is worn no lameness is apparent. A most interesting fea-

Johnston: Fig. 3.—After Recovery.

Case III.

Johnston: Fig. 5.

Anterior view of left leg, showing false articulation seven years after exsection of fibia.

ture of the skiagraph is the exhibition of a false joint in the new tibia about three inches above the ankle-joint.

Case IV.—A boy aged 12 years was seized with pain in his shin. This was soon followed by redness and swelling. The trouble was at first ascribed to rheumatism. Later Dr. Warinner was called and pronounced it osteomyelitis. I first examined him at the Old Dominion Hospital. His leg was enormously swollen and very red, and fluctuation was present throughout the entire extent of the tibia. The boy being profoundly septic, and his condition being alarming, operation was immediately performed.

An incision was made extending the whole length of the tibia. When the bone was exposed it was discovered to be entirely denuded of its periosteum, and, like Case III., was lying loose in a pus sac. It was simply lifted out without any resistance by adherent membranes or epiphyses. As in the other cases, the wound was cleansed and dressed antiseptically and the leg placed in a fracture-box.

Prompt relief from constitutional symptoms supervened and the boy's general health rapidly improved. He made excellent progress and was able leave at the end of eight weeks with a light plaster-of-Paris dressing. He was allowed to go on crutches, with the leg suspended, at the end of ten weeks. In six months the bone, which was much distorted, was able to bear the weight of his body.

It will be seen, by reference to the skiagraph of this case, that, unlike Case II., formation of the bone was incomplete, there being a hiatus of fibrous tissue about the middle of the bone. The leg is sufficiently strong, however, to bear the weight of the body without the aid even of a cane. In consequence of the removal of the entire shaft and the destruction of the epiphyseal junction there is, of course, much shortening of the new tibia, but by means of a high shoe there is but little lameness. The function of the limb is excellent.

Case V.—L. J., a boy, aged 11 years, referred by Dr. H. H. Henry, was admitted to the Old Dominion Hospital, January 5, 1903. At the time of his admission his condition was deplorable. He had an acute osteomyelitis of the left tibia and a very acute inflammation in the right hip-joint. He was so profoundly septic that his life was despaired of. His symptoms had developed with great rapidity and violence, following a slight hurt. The whole of the left leg was very red and enormously swollen. Fluctuation was present over the whole tibial region.

An incision was made the entire length of the shin-bone, which was found suspended in a pus sac, and, like Cases III. and IV., the bone was free. No force was required to extract it, it being literally lifted out of its bed. This case was treated

Case IV.

Johnston: Fig. 6.—Skiagraph of Case IV.

like the others, and at the same time treatment was applied to the diseased hip-joint on the opposite side. The issue in this case appeared doubtful for many days, on account of the profound sepsis present, but finally the little fellow began to improve, and, after a long and tedious illness, he recovered, with an excellent new tibia in the left leg and a good result from treatment

Case IV.

Johnston: Fig. 7.—After Recovery.

of hip-joint disease of the other side. The after-treatment of this case was the same as in the previous cases, including the fracture-box. After the new bone had formed and the wound completely cicatrised, a plaster-of-Paris cast was applied for the protection of the new bone and to give rigidity to the limb. At the end of ten weeks the boy was able to go on crutches, and later, by the aid of a high-soled shoe, could walk without great lameness, and what lameness was present arose from the injury to the right hip.

I regret not being able to present a skiagraph of this case, but an excellent photograph of the injured leg is shown.

This boy lived comfortably and was able to get about in excellent shape for several months. In the spring of 1903, he was

CASE V.

JOHNSTON: FIG. 8.—AFTER RECOVERY.

seized with some acute abdominal trouble, supposedly an abscess of the mesentery, from which he died.

CASE VI.—H. T., a sturdy boy, aged 5 years, was referred by his uncle, Dr. Henry Turnbull, of Lawrenceville. This little fellow came in one evening from play and complained to his mother of pain in his leg, over the fibula, saying he wanted to go to sleep. His mother examined his leg and found the ankle a little red and somewhat swollen. The next morning the leg was very much swollen, and there was high fever. He suffered greatly and was not able to walk, and the limb remained in this swollen condition for five or six weeks, when a "soft place" was

CASE VI.

JOHNSTON: FIG. 9.

Photograph eight years after operation.

noticed on the ankle, and the attending physician opened this and kept it open. In two weeks small particles of bone worked out of the wound. At this juncture I saw him.

I discovered that the major portion of the shaft of the fibula was necrotic. Incision over the bone was made, extending upward from the external malleolus to within three inches of the head of the fibula. It was manifest that no part of the bone could be

Case VI.

Johnston: Fig. 10.—Skiagraph of Case VI.

saved except the lower malleolus and a small portion of the upper extremity.

With a Gigli saw the bone was divided through its healthy parts and the diseased portion removed. With the free drainage thus accomplished and the removal of the necrotic bone, septic symptoms quickly subsided. The after-treatment was precisely as in the others, viz., antiseptic dressings and immobilisation in a fracture-box. This little fellow did remarkably well, and in six weeks was able to leave the hospital with his leg in a plaster-of-Paris dressing. The bone rapidly reformed, and in six months the child was entirely restored to health, with the limb unimpaired. Eight years after operation the accompanying photograph (Fig. 9) of the leg and a skiagraph of the bone were made. It will be seen, from the skiagraph, that the bone is perfect. The leg is absolutely perfect in function, and the youth, now thirteen, is as sturdy and healthy as any boy of his age.

In considering these cases I am impressed with a number of facts well worth emphasising:

1. All my cases were in males.
2. In every instance they were in growing children.
3. The trouble was always traceable to a trivial injury.
4. Exposed bones were involved: the tibia in five instances, the fibula in one.
5. There was remarkable variation in the degree of virulence of infection.
6. The extent of destruction was dependent upon the character of the infection.
7. There were profound constitutional symptoms where the infection was virulent.
8. The condition was often mistaken for "growing pains," rheumatism, or erysipelas.
9. Regeneration in my cases was entirely of periosteal origin, and was rapid and complete.
10. Deformity resulted only in such cases as had suffered destruction of the epiphyseal line.
11. There is no deformity where a disk of bone is left between the shaft and the epiphysis.
12. Invariably the companion bone takes on compensatory hypertrophy.
13. Time to operate:
    (*a*) In acute cases (imperative) immediately.
    (*b*) In subacute or chronic cases (elective) when new bone tissue has begun to appear.
14. Operation:
    (*a*) Free incision and complete removal of *all* diseased bone.

(*b*) Spare all periosteum possible.
(*c*) Avoid curet, or use cautiously.
(*d*) Purify the wound by the strictest antiseptic methods

After treatment:

(*a*) Maintain aseptic conditions.
(*b*) Avoid too frequent and rough dressings.
(*c*) Treat as a fracture, by immobilisation in a fracture-box.
(*d*) Carefully shape the parts, as bone tissue develops, by bandages or adhesive straps.
(*e*) Protect the young bone by means of plaster-of-Paris.
(*f*) Abstain from use of the limb until the new bone is capable of sustaining the weight of the body.
(*g*) Look after general health.

407 East Grace Street.

## Figures and Tables

**Fig. 1. f1:**
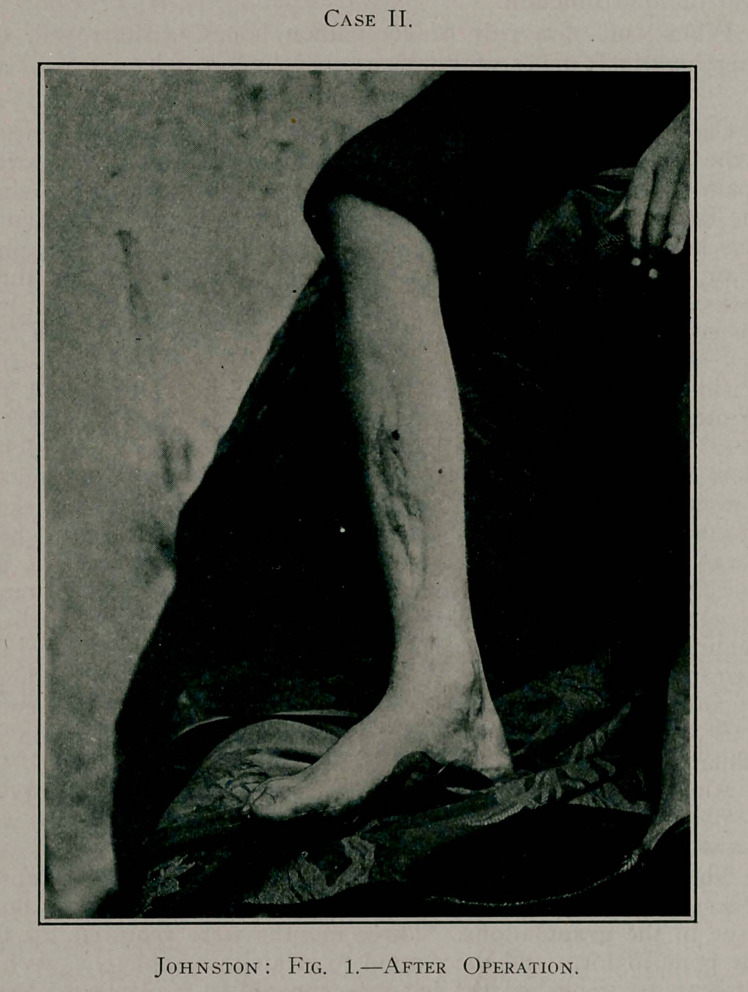


**Fig. 2. f2:**
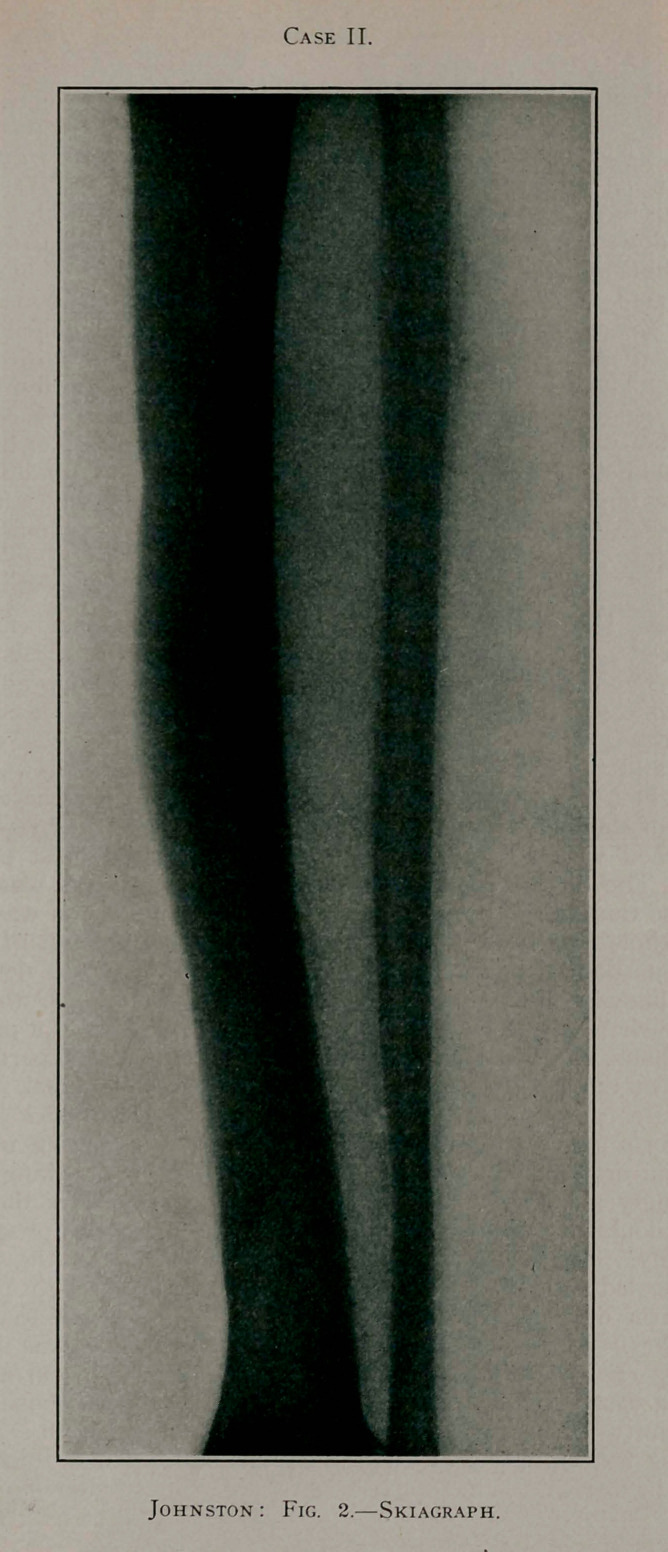


**Fig. 3. f3:**
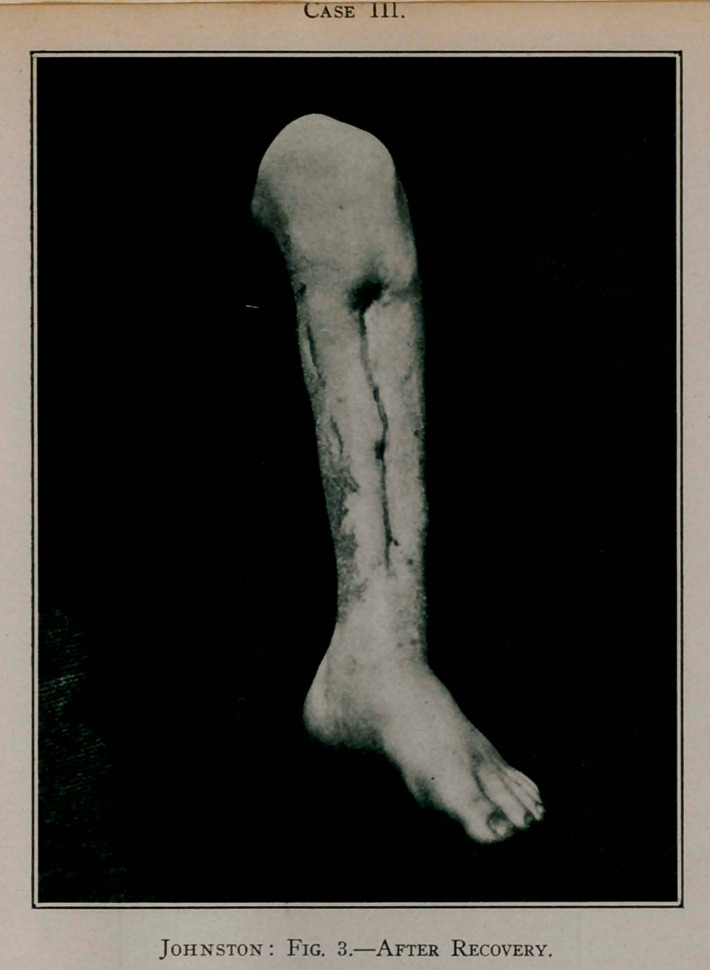


**Figure f4:**
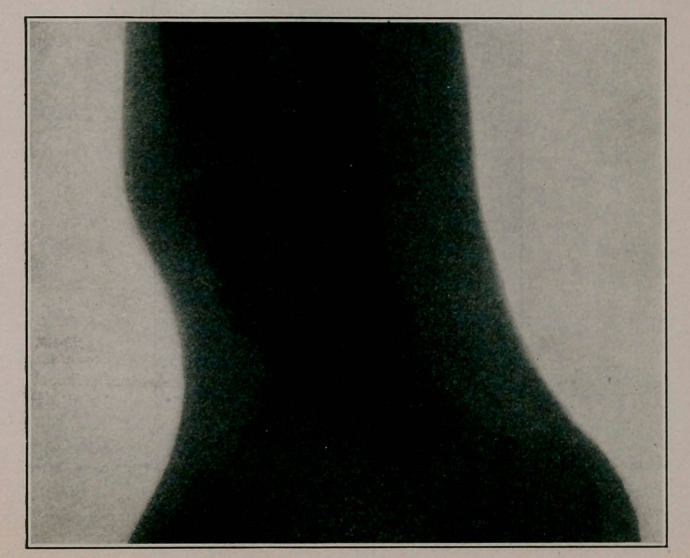


**Fig. 5. f5:**
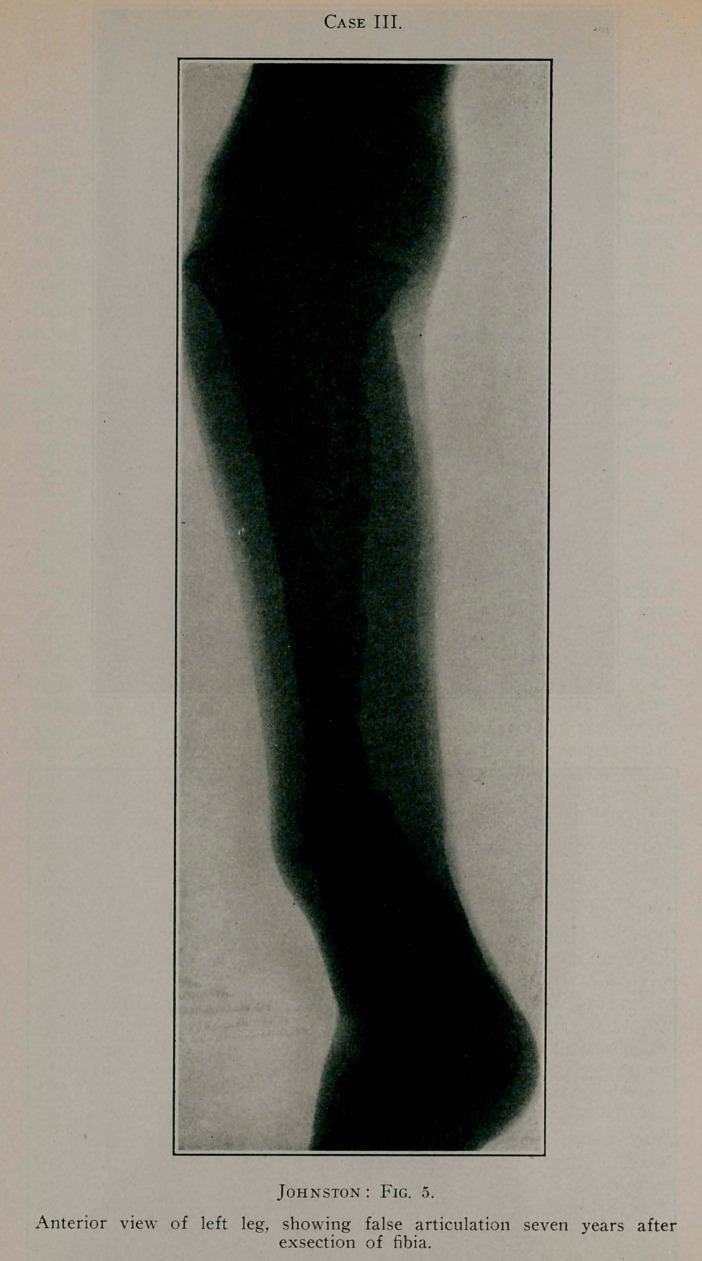


**Fig. 6. f6:**
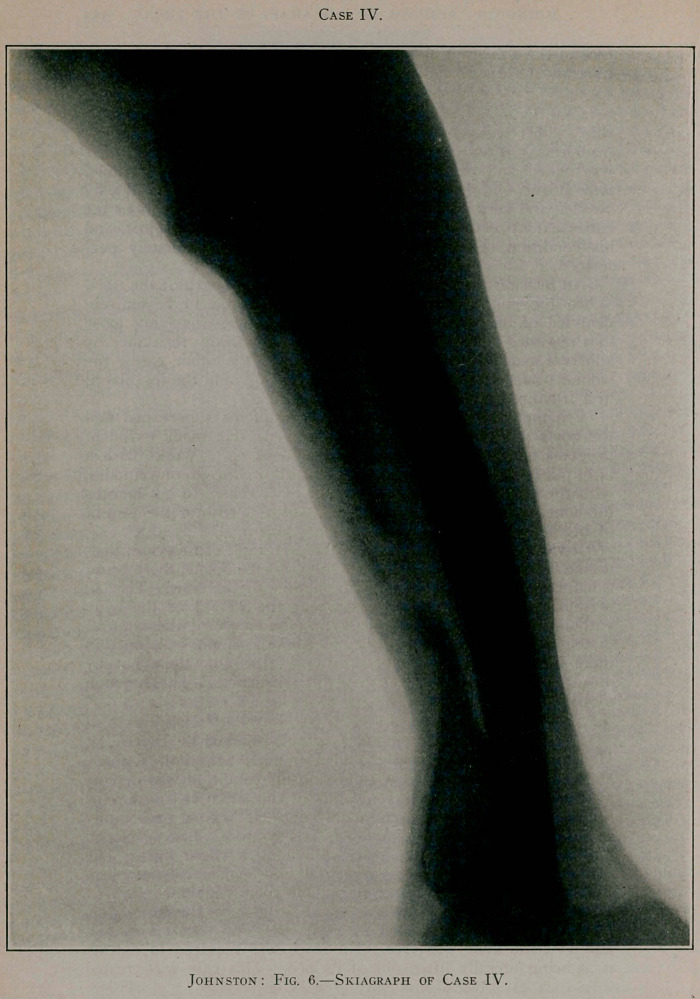


**Fig. 7. f7:**
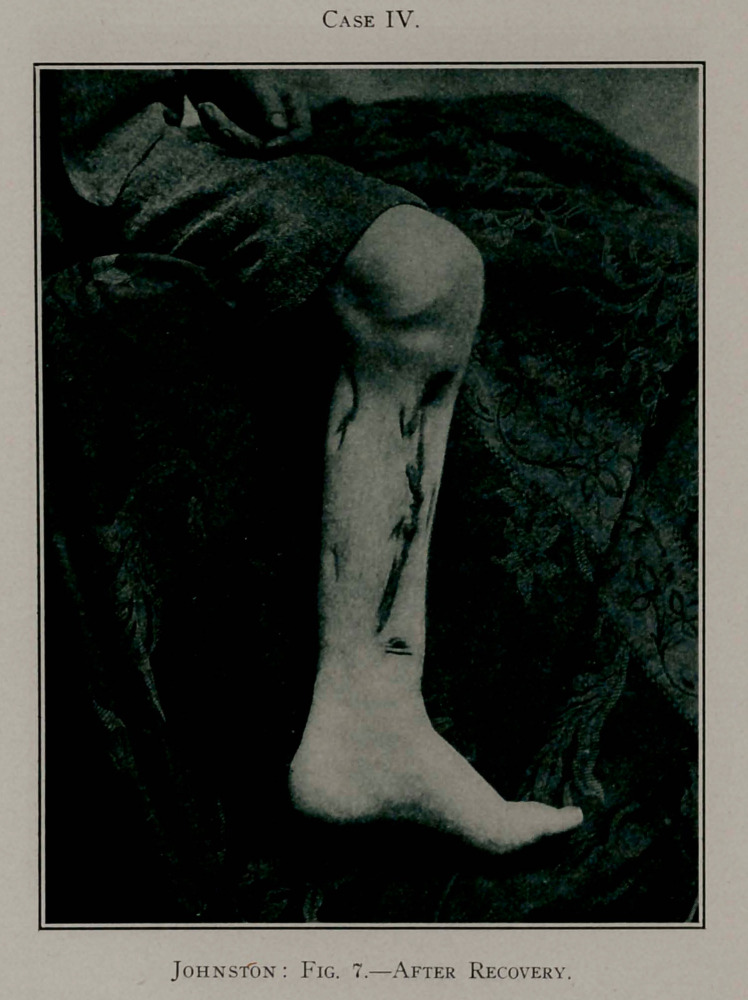


**Fig. 8. f8:**
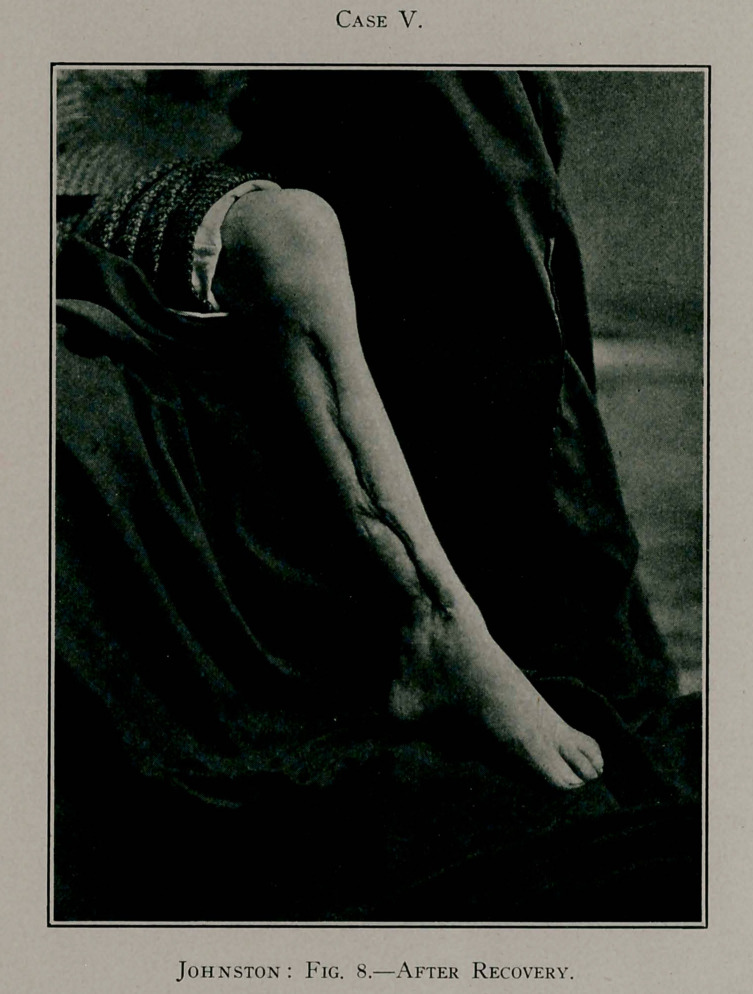


**Fig. 9. f9:**
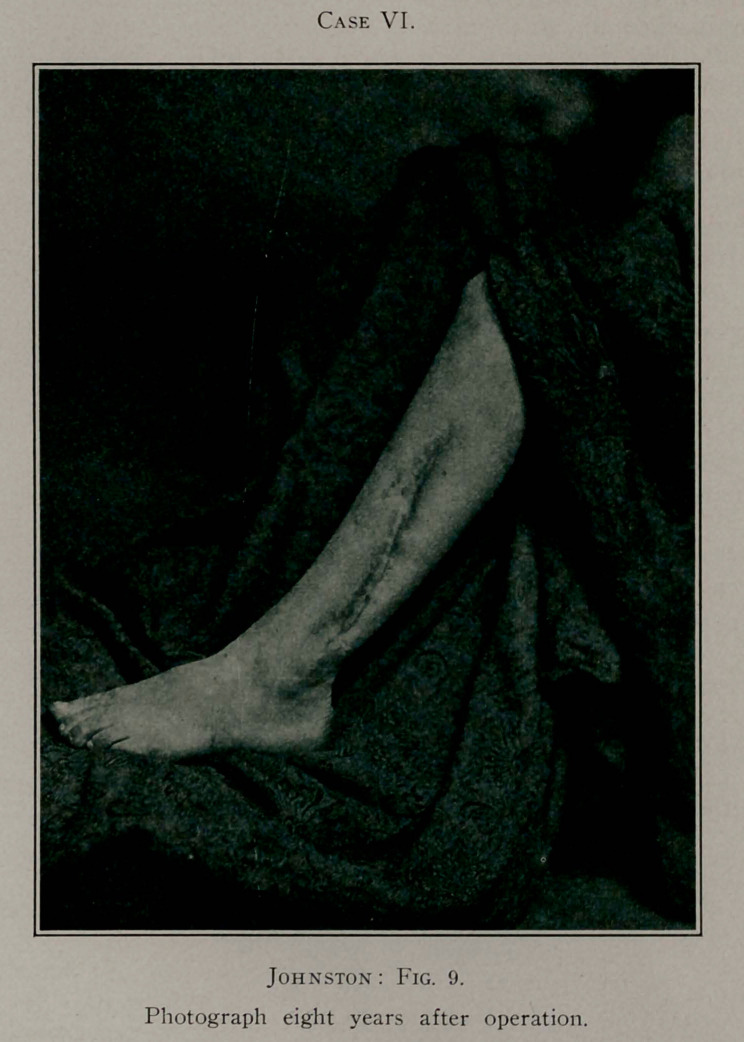


**Fig. 10. f10:**